# 3D Printed Scaffold Based on Type I Collagen/PLGA_TGF-β1 Nanoparticles Mimicking the Growth Factor Footprint of Human Bone Tissue

**DOI:** 10.3390/polym14050857

**Published:** 2022-02-22

**Authors:** Federica Banche-Niclot, Caterina Licini, Giorgia Montalbano, Sonia Fiorilli, Monica Mattioli-Belmonte, Chiara Vitale-Brovarone

**Affiliations:** 1 Department of Applied Science and Technology, Politecnico di Torino, 10129 Torino, Italy; federica.banche@polito.it (F.B.-N.); giorgia.montalbano@polito.it (G.M.); sonia.fiorilli@polito.it (S.F.); 2 Department of Clinical and Molecular Sciences (DISCLIMO), Università Politecnica delle Marche, 60126 Ancona, Italy; c.licini@pm.univpm.it (C.L.); m.mattioli@univpm.it (M.M.-B.); 3 National Interuniversity Consortium of Materials Science and Technology, RU Politecnico di Torino, 50121 Firenze, Italy

**Keywords:** drug delivery, polymeric nanoparticles, TGF-β1, type I collagen, 3D printed scaffolds, bone, tissue regeneration

## Abstract

In bone regenerative strategies, the controlled release of growth factors is one of the main aspects for successful tissue regeneration. Recent trends in the drug delivery field increased the interest in the development of biodegradable systems able to protect and transport active agents. In the present study, we designed degradable poly(lactic-co-glycolic)acid (PLGA) nanocarriers suitable for the release of Transforming Growth Factor-beta 1 (TGF-β1), a key molecule in the management of bone cells behaviour. Spherical TGF-β1-containing PLGA (PLGA_TGF-β1) nanoparticles (ca.250 nm) exhibiting high encapsulation efficiency (ca.64%) were successfully synthesized. The TGF-β1 nanocarriers were subsequently combined with type I collagen for the fabrication of nanostructured 3D printed scaffolds able to mimic the TGF-β1 presence in the human bone extracellular matrix (ECM). The homogeneous hybrid formulation underwent a comprehensive rheological characterisation in view of 3D printing. The 3D printed collagen-based scaffolds (10 mm × 10 mm × 1 mm) successfully mimicked the TGF-β1 presence in human bone ECM as assessed by immunohistochemical TGF-β1 staining, covering ca.3.4% of the whole scaffold area. Moreover, the collagenous matrix was able to reduce the initial burst release observed in the first 24 h from about 38% for the PLGA_TGF-β1 alone to 14.5%, proving that the nanocarriers incorporation into collagen allows achieving sustained release kinetics.

## 1. Introduction

Bone is a dynamic tissue that undergoes a lifelong process, known as remodelling, where bone resorption operated by osteoclasts (OCs) and new bone deposition through osteoblasts (OBs) are harmonically coupled. During the resorption phase, OCs create a local acidic environment and secrete enzymes onto the underlying extracellular matrix (ECM), resulting in the solubilization of the nanohydroxyapatite crystals, the degradation of collagenous fibres, and the consequent release of stored biomolecules, such as growth factors (GFs) [1], that are soluble proteins responsible for regulating several cellular processes [2].

One of the most abundant GFs in the bone tissue is Transforming Growth Factor-beta 1 (TGF-β1), a 25 kDa protein belonging to the TGF-β superfamily [3]. TGF-β1 is stored in bone ECM as inactive form combined in a Large Latent Complex (LLC) to enhance the protein stability and prevent protein-receptors bound [4,5]. During the bone resorption, enzymes secreted by OCs as Matrix Metalloproteases (MMPs) enable the release and subsequent activation of latent TGF-β1 by cleavage of the LLC [6]. The amount of available protein during the different phases of bone remodelling has an impact on the different TGF-β1 functions: low levels induce OBs-precursor migration and differentiation towards mature OBs at the resorptive site, as well as the increase of OBs proliferation while exerting an anti-apoptotic role; high levels of TGF-β1 inhibit OBs proliferation, stimulating protein synthesis, mainly type I collagen [7,8,9,10,11,12]. Moreover, a low amount of TGF-β1 promotes OCs differentiation, which is repressed in case of high protein levels [9,12,13,14].

Despite its key role and several investigations assessing the effect of free TGF-β1 on bone tissue regeneration [7,9,15,16] a limited number of studies evaluated TGF-β1 expression in the bone matrix [6,17,18], and to date, few have been focused on its localization and distribution within bone ECM [19]. TGF-β1 native form, however, presents poor stability with a short effective half-life, and conventional administration methods (e.g., parenteral, oral) result in systemic distribution of the therapeutic agents, with an ineffective targeting. Moreover, to maintain a useful protein concentration at the site of action all along, multiple and/or high-dose administrations are required with consequent side effects [20,21,22,23]. Consequently, strategies for its local delivery to maintain a useful protein concentration at the site of action all along are desirable.

Over the last decades, scientists focused their attention on the development of long-term drug delivery systems (DDS) that would overcome these limitations, providing a controlled and sustained release of biomolecules at the lesion site and being beneficial for the induction of bone regeneration [24,25]. This engineering approach includes the design of nano- and microcarriers based on different systems like liposomes [26,27,28], dendrimers [29,30,31], and polymeric particles that, due to their physico-chemical properties, can protect the incorporated biomolecules from premature degradation, enhancing their bioavailability, bioactivity, and biodistribution [32,33]. Since the first studies conducted by Langer and Folkman in 1976, the design of DDS using biodegradable synthetic polymer-based nanoparticles (NPs) gained increasing consideration as a vehicle for proteins and peptides, including GFs, thanks to their excellent biocompatibility, high surface-to-volume ratio, high loading efficiency and stimuli-response behaviour [34,35]. Among the synthetic polymers, poly(lactic-co-glycolic) acid (PLGA) is one of the most promising materials for biomedical and pharmaceutical applications (including NPs) due to the proven in vivo biocompatibility (PLGA devices are approved by FDA and EMA), minimal systemic toxicity and tuneable biodegradability [36,37,38]. PLGA-based NPs can increase the stability and protect the loaded biomolecules from enzymatic degradation, providing a satisfactory payload delivery with the appropriate therapeutic duration, biodistribution and concentration [39,40]. Typically, the release of encapsulated bioactive molecules from these biodegradable nanocarriers is regulated by the diffusion of the molecules throughout the polymer matrix, followed by the bulk erosion of the material itself [41,42,43,44].

In addition, to avoid the premature release of incorporated biomolecules and GFs while providing a more targeted and sustained release to support bone regeneration, these NPs can be further integrated into biocompatible and biodegradable polymeric-based scaffolds [45,46]. These structures may be designed using either natural polymers such as alginate [47,48,49,50], collagen [17,51,52,53,54,55,56], gelatin [57,58,59,60], and fibrin [61,62,63], or synthetic polymers like polycaprolactone (PCL) [64,65] and polyurethanes (PU) [66,67,68]. Compared to synthetic materials, naturally derived polymers generally show superior biocompatibility and bioactivity although they lack mechanical strength and appropriate degradation rates. As the main organic phase of the natural bone ECM, type I collagen represents one of the most extensively used natural polymers in tissue engineering. This protein plays an important role in bone structural and mechanical features, as well as cell attachment, migration, and organization [69,70]. Thanks to the ability of collagen molecules to self-assemble into fibrils under physiological conditions, collagen-based systems can form physically crosslinked hydrogels when exposed at 37 °C [71]. Besides, collagen-based biomaterials can induce mineralization and support osteogenic differentiation [72,73,74].

Based on these considerations, the use of two different biocompatible polymers, i.e., PLGA and type I collagen was explored to design a smart biomimetic delivery platform able to modulate the release of TGF-β1 while mimicking the GF bone content and distribution. As the first goal of the present study, the feasibility of using biodegradable PLGA NPs as carriers for the encapsulation and the subsequent delivery of TGF-β1 was investigated. With the aim to reproduce the TGF-β1 amount and distribution detected in human bone ECM, an innovative composite system was developed by combining TGF-β1-containing PLGA NPs (PLGA_TGF-β1) with type I collagen for the fabrication of a nanostructured 3D printed scaffold capable of providing a sustained release of the GF. With this perspective, the hybrid formulation was firstly characterised for its suitability as biomaterial ink, and the further localisation of TGF-β1 into the resulting 3D constructs was explored using histochemical analyses.

To the best of the authors’ knowledge, this is the first study in the bone tissue engineering area that reported the integration of GF-containing nanocarriers into a collagen-based 3D-printed scaffold with the challenging aim to mimic the GFs content, spatial distribution and release occurring in natural bone ECM. The development of 3D constructs characterised by a biomimetic distribution of biomolecules can facilitate cellular recognition, further enhancing the efficacy and the therapeutic effects of the encapsulated GF. Moreover, the strategy developed and presented in this study, which combines the use of versatile PLGA particles able to encapsulate various drugs and proteins with the 3D printing of soft polymeric systems, can be exploited in different research fields and may lead to the realization of implantable devices applicable in personalized treatments.

## 2. Materials and Methods

### 2.1. TGF-β1 Expression in Bone ECM

#### 2.1.1. Sample Collection

Bone samples were obtained from three healthy humeral heads discarded during prosthetic replacement of the shoulder prosthesis surgery carried out at Istituto Ortopedico Rizzoli (IOR), Under the Local Ethical Committee approval (protocol code 0008296, 11 April 2016) and with the 1964 Helsinki declaration, informed consent was obtained. The patients were aware that the tissue used for the study represented a discard from the surgical procedure and voluntarily participated in the study (freedom from coercion or undue influence, real or imagined). The humeral heads were deprived of most of the soft tissue, washed in Phosphate Buffered Saline (PBS) pH 7.4, dried, and stored at −80 °C until use.

#### 2.1.2. Immunohistochemistry

The humeral heads were cut, and the obtained fragments were fixed in 4% neutral buffered formaldehyde solution for 48 h, decalcified by Biodec R (Bio-Optica, Milan, Italy) for 6 h, and then routinely processed for paraffin embedding at temperatures not exceeding 56 °C. For immunohistochemical analysis, 6-μm-thick paraffin-embedded tissue sections were deparaffinized and rehydrated with xylene and a graded series of ethyl alcohols (from 100% to 50%), before incubation with 3% hydrogen peroxide for 30 min to block endogenous peroxidase activity. Antigen retrieval was performed in 0.3% Tween 20 in PBS 1× at room temperature (RT) for 20 min. Sections were then incubated overnight at 4 °C with anti-TGF-β1 antibody (dilution 1:200; GeneTex, Irvine, CA, USA). Antigen was visualized by Envision Dako REAL™ EnVision™ Detection System (Dako, Santa Clara, CA, USA). Sections were counterstained with Mayer’s haematoxylin, dehydrated, and mounted in Biomount HM (Bio-Optica, Milan, Italy).

#### 2.1.3. Immunostaining Evaluation

The expression level of TGF-β1 in bone ECM was evaluated as area percentage in a semi-quantitative manner. Six different images from each sample (20× magnification) were examined by ImageJ software (https://imagej.nih.gov/ij/, accessed on 28 May 2020). The staining was measured after colour deconvolution, to obtain only the specific stain and calculated on bone ECM area, selected as Region of Interest (ROI), excluding osteocyte lacunae and endosteum.

#### 2.1.4. Protein Extraction and TGF-β1 ELISA Quantitation

Bone samples (100 mg) were obtained using a hammer and chisel, immersed in a saline solution pH 7.2 with protease inhibitors (Sigma-Aldrich, Milan, Italy), sonicated in an ultrasonic bath for 1 min, and interposed with 1 min in ice, for 5 cycles overall, to degrease and remove the remaining soft tissues and cells. Samples were then powdered in liquid nitrogen and total protein extraction was performed by TRIzol reagent (Invitrogen, Carlsbad, CA, USA), according to the manufacturer’s recommended protocol. Total protein amount was assessed by BCA assay kit (Thermo Scientific, Milan, Italy) and used to normalize the results. All protein extracts were acidified by 1 M HCl to activate latent TGF-β1, and then neutralized by 1.2 M NaOH/0.5 M HEPES. Bone ECM TGF-β1 concentration was assessed by a commercial enzyme-linked immunosorbent assay (ELISA) kit (Cloud-Clone Corp., Katy, TX, USA). The measurements were executed in duplicate, according to the manual’s instructions.

### 2.2. PLGA Nanoparticles Containing TGF-β1 (PLGA_TGF-β1)

#### 2.2.1. Synthesis of TGF-β1-Containing PLGA Nanoparticles

**Figure 1:**
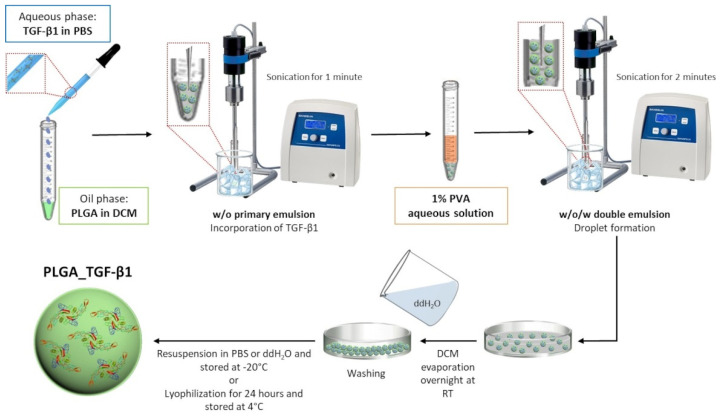
Schematic representation of PLGA_TGF-β1 synthesis process.

TGF-β1-containing PLGA (PLGA_TGF-β1) nanoparticles (NPs) were prepared by water-in-oil-in-water (w/o/w) double-emulsion method followed by solvent evaporation [75]. A schematic representation of the synthesis technique is reported in Figure 1. Briefly, an aqueous solution containing recombinant human transforming growth factor-β1 (TGF-β1; Sigma-Aldrich, Milan, Italy) (100 ng/mL) was emulsified into the organic phase, consisting of poly(DL-lactic-co-glycolic acid) (PLGA 50:50, Mw 25,000; Sigma-Aldrich, Milan, Italy) dissolved in dichloromethane (DCM; Sigma-Aldrich, Milan, Italy), using a probe sonicator (Sonoplus GM3200, Bandelin, Berlin, Germany) for 1 min on ice (20% amplitude), to yield a water-in-oil emulsion and allow TGF-β1 incorporation. Subsequently, 5 mL of 1% poly(vinyl alcohol) (PVA, 87–90% hydrolyzed; Sigma-Aldrich, Milan, Italy) aqueous solution was added to the primary emulsion and further emulsified by sonication on ice for 2 min at 20% amplitude to stabilize the droplet formation. The organic solvent was then allowed to evaporate overnight at RT. PLGA_TGF-β1 nanoparticles were collected by centrifugation and washed several times with double-distilled water (ddH_2_O) and finally resuspended in PBS or ddH_2_O (depending on further analyses) and stored at −20 °C for long-term storage or lyophilized (Lyovapor L-200 freeze-dryer, Büchi, Cornaredo, Italy) over 24 h for further analysis. Unloaded PLGA nanoparticles (PLGA_Ø) were used as control and were prepared following the method described above, without adding TGF-β1 to the aqueous solution.

#### 2.2.2. Characterisation of PLGA_TGF-β1 Nanocarriers

The morphology of lyophilized nanoparticles was studied by means of Field-Emission Scanning Electron Microscope (FESEM; ZEISS MERLIN instrument, Oberkochen, Germany) at 3.0 kV after platinum sputter coating. In order to better evaluate the morphology of PLGA_TGF-β1, one droplet of the aqueous suspension containing nanoparticles was placed on a copper grid with carbon film (3.05 mm in diameter, 200 mesh; TAAB, Aldermaston, UK). The sample was dried and coated with platinum before analysis to increase sample conductivity. The NPs were characterized in terms of size, size distribution and zeta potential by using a dynamic light scattering (DLS) measurement instrument (Zetasizer Nano ZS90, Malvern Instruments Inc., Malvern, UK). For the evaluation of size and polydispersity index (PDI), a suspension of PLGA_TGF-β1 in ddH_2_O at a concentration of 0.1 mg/mL was obtained. Next, 1 mL of suspension was placed in a polystyrene cuvette and 15 runs for 3 measures were performed. The zeta potential was evaluated with a disposable capillary cell having a volume of 1 mL, performing 100 runs in triplicate. All the analyses were performed at 25 °C. The viscosity, refraction index and dielectric constant were set to the reference values of water.

#### 2.2.3. Efficiency of TGF-β1 Encapsulation into the Polymeric Nanocarriers

The amount of TGF-β1 encapsulated was determined after extraction from PLGA nanoparticles, following procedures reported in the literature [76]. Briefly, a predefined amount of dry PLGA_TGF-β1 was dissolved in 4 mL of 0.1 M NaOH containing 0.5% (*w*/*v*) Sodium Dodecyl Sulphate (SDS) by stirring for 3 h at 37 °C. After centrifuging at 10,000 rpm for 10 min (Hermle Labortechnik Z326, Wehingen, Germany), the aqueous solution was collected and the TGF-β1 content was quantified using a human TGF-β1 Quantikine^®^ ELISA kit (R&D Systems, Minneapolis, MN, USA) according to the manufacturer’s instructions. Experiment and analysis were conducted in duplicate. The percentage of encapsulation efficiency was expressed as the ratio of the actual value of total protein content to the theoretical value. Furthermore, the indirect encapsulation efficiency was calculated by evaluating the presence of TGF-β1 in the aqueous washing supernatant collected. The encapsulation efficiency (EE%) and indirect encapsulation efficiency (iEE%) of TGF-β1 were calculated as follows:

(1) $$\mathrm{EE}\%=\frac{\mathrm{amount}\;\mathrm{of}\;\mathrm{incorporated}\;\mathrm{TGF}-\beta 1}{\mathrm{initial}\;\mathrm{amount}\;\mathrm{of}\;\mathrm{TGF}-\beta 1}\%$$

(2) $$\mathrm{iEE}=\frac{\left(\mathrm{initial}\;\mathrm{amount}\;\mathrm{of}\;\mathrm{TGF}-\beta 1\right)-\left(\mathrm{amount}\;\mathrm{of}\;\mathrm{TGF}-\beta 1\;\mathrm{lost}\;\mathrm{during}\;\mathrm{washing}\right)}{\mathrm{initial}\;\mathrm{amount}\;\mathrm{of}\;\mathrm{TGF}-\beta 1}\%$$

ELISA assay on empty samples (PLGA_Ø) was used as negative control and subtracted.

#### 2.2.4. In Vitro TGF-β1 Release Kinetic from PLGA NPs

In order to evaluate the TGF-β1 release kinetic, PLGA_ TGF-β1 NPs were soaked in PBS (pH 7.4) at a concentration of 10 mg/mL. The suspension was placed in an orbital shaker (Excella E24, Eppendorf, Hamburg, Germany) at 37 °C, 70 rpm for up to 28 days. Experiments were conducted in triplicate. At each time point, samples were centrifuged, the entire volume was withdrawn and replaced with an equal amount of fresh release media. The collected samples were stored at −20 °C until analysis. The concentration of TGF-β1 in the collected release media was determined using human TGF-β1 Quantikine^®^ ELISA Assay (R&D Systems, Minneapolis, MN, USA). The measurements were performed in duplicate, and the cumulative percentages of released TGF-β1 were reported as mean ± standard deviation.

### 2.3. Collagen-Based 3D Printed Scaffolds

#### 2.3.1. Preparation of the PLGA_TGF-β1 Containing Collagenous Formulation

With the ambitious aim to fabricate 3D scaffolds containing the same amount of TGF-β1 with a similar localisation observed in the human bone ECM, PLGA_TGF-β1 nanocarriers were incorporated into 1.5% type I collagen solution. The amount of TGF-β1 introduced in the collagenous formulation was calculated according to the area percentage calculated by semi-quantitative evaluation of immunostaining (Section Immunostaining evaluation).

Type I collagen powder (type I collagen from bovine Achilles tendon; Blafar Ltd., Dublin, Ireland) was dissolved in 0.5 M acetic acid solution under overnight stirring at 4 °C. The pH of the solution was then neutralised by adding 1 M NaOH reaching a final collagen concentration of 1.5 wt% [77]. Considering the EE% of the synthesised nanocarriers, the calculated amount of PLGA_TGF-β1 NPs was dispersed in PBS, pH 7.4 and subsequently added to the collagen solution to obtain a homogeneous dispersion of the NPs in the final formulation. The resulting suspension, named Coll/PLGA_TGF-β1, was further stirred at 4 °C in order to guarantee optimal homogeneity and then stored at 4 °C until use. The entire process was carried out at 4 °C to avoid the premature gelation of the collagen-based suspension.

#### 2.3.2. Rheological Tests

A DHR-2 controlled stress rotational rheometer (TA Instruments, Waters, Milan, Italy) was used to carry out the rheological analysis on the Coll/PLGA_TGF-β1 system before and after the collagen sol–gel transition at 37 °C. All tests were conducted using a parallel plate geometry with a diameter of 20 mm and the temperature of the system was continuously controlled by means of a Peltier plate system. Flow ramp tests at 10 °C were conducted to investigate the variation in the suspension viscosity over a wide range of shear rates (0.01–1000 s^−1^) while the sol–gel transition of the system was observed by means of a time sweep analysis carried out at 37 °C under 1% strain for 60 min. The visco-elastic properties of the Coll/PLGA_TGF-β1 system before and after the genipin chemical crosslinking was assessed on samples measuring 20 mm in diameter, through dynamic amplitude sweep tests (0.01–1% strain, 37 °C and 1 Hz) while the thermal stability of the composite material was explored by means of dynamic temperature ramp tests (1% strain; 1 Hz) up to 80 °C using a heating rate of 5 °C/min.

#### 2.3.3. Scaffold Manufacturing

**Figure 2:**
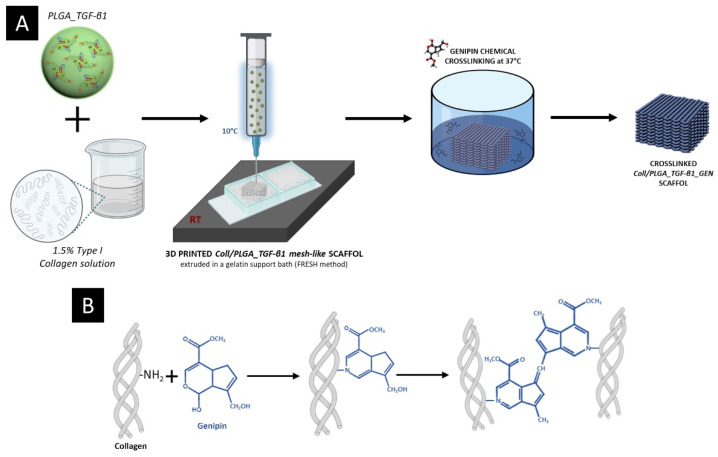
(**A**) Schematic representation of Coll/PLGA_TGF-β1_GEN scaffold realization process and (**B**) mechanism of genipin chemical crosslinking of collagen.

Coll/PLGA_TGF-β1 suspensions were processed using a commercial bioprinter (BioX, CELLINK, Gothenburg, Sweden) equipped with a pneumatic temperature-controlled print-head and using 27 G (200-micron internal diameter) needles. The suspension was loaded in a 3 mL cartridge kept at 10 °C and extruded in supporting gelatin (powder, Type A; Sigma-Aldrich, St. Louis, MO, USA) slurry kept at 20 °C and obtained following the procedure reported by Hinton and coworkers [78]. Mesh-like structures (10 mm × 10 mm area, 1 mm thickness) were printed at a pressure of 50 kPa, printhead speed of 15 mm/s, and a z layer of 140 µm, considering an infill percentage of 15%. After printing, the scaffolds were incubated at 37 °C for 3 h to promote the gelation of the system and the removal of the gelatin bath. The collected mesh-like structures were washed in ddH_2_O to remove any gelatin residuals and further processed with genipin solutions to induce the chemical crosslinking of collagen. In detail, scaffolds were incubated in a 0.5% genipin (powder; Challenge Bioproducts, Yun-Lin Hsien, Taiwan) solution in 70% ethanol (GEN/EtOH) at 37 °C for 24 h, following a procedure previously optimized by the authors [17]. After the chemical crosslinking, the scaffolds (Coll/PLGA_TGF-β1_GEN) were washed with ddH_2_O to remove the genipin residuals and collected for further analyses. A schematic representation of the process for the realization of the composite scaffolds is reported in Figure 2.

#### 2.3.4. Morphological and Physico-Chemical Assessment of Coll/PLGA_TGF-β1_GEN Scaffolds

The obtained scaffolds were lyophilized and subsequently sectioned to examine the micro- and nanostructure of the composite material as well as the NPs distribution by means of FESEM. For this analysis, samples were freeze-dried for 24 h, then sputter-coated with platinum and examined at 1.5 kV and 3 kV. Fourier transform infrared spectroscopy in attenuated total reflectance mode (ATR-FTIR) was performed to assess the effective NPs incorporation and confirm the chemical composition of the composite material. The physico-chemical assessment of the hybrid scaffolds was conducted on both crosslinked and non-crosslinked samples after freezing at −20 °C and lyophilization for 24 h. ATR-FTIR spectra were collected between 4000 and 600 cm^−1^ at 4 cm^−1^ resolution using 32 scans using an Equinox 55 spectrometer (Bruker, Ettlingen, Germany) equipped with an MCT cryodetector and ATR accessory. The spectra were reported after background subtraction, baseline correction and smoothing (11 points) using OPUS software (Bruker, Ettlingen, Germany).

#### 2.3.5. TGF-β1 Release Test from the Composite System

The TGF-β1 release kinetics from the crosslinked Coll/PLGA_TGF-β1_GEN scaffolds was studied at 37 °C over 28 days. After the incubation in GEN/EtOH for 24 h, Coll/PLGA_TGF-β1 scaffolds were immersed in 2 mL of PBS, under a constant agitation of 70 rpm at 37 °C up to 28 days. At indicated time points, the entire volume of PBS was collected, stored at −20 °C until analysis and replaced by a fresh buffer. The released quantity of TGF-β1 was determined by human TGF-β1 ELISA Assay (R&D Systems, Minneapolis, MN, USA) according to the manufacturer’s instructions. Analyses were conducted in triplicate and the percentages of released TGF-β1 are reported as mean ± standard deviation.

#### 2.3.6. Histochemical Assessment of PLGA_TGF-β1 NPs Distribution in the 3D Printed Collagen-Based Scaffolds

To perform the histochemical assessment, scaffolds were fixed in 4% formaldehyde solution in PBS (Santa Cruz Biotechnology, Dallas, TX, USA) for 30 min and, washed in PBS 1× twice. Samples were then dehydrated by a graded series of ethyl alcohols (from 50% to 100%), clarified by xylene for 45 min twice and incubated in paraffin overnight at 56 °C to allow paraffin infiltration. After two incubations in paraffin, samples were embedded in paraffin blocks horizontally. Ten-micrometre-thick slices were cut and, after deparaffination and rehydration, Haematoxylin and Eosin and Sirius Red staining were performed. Haematoxylin and Eosin staining was observed by light microscope, while Sirius Red staining by both light and fluorescence microscope. The homogeneity PLGA_TGF-β1 NPs distribution throughout the composite scaffold was investigated by immunohistochemistry on 10-µm-thick paraffin-embedded sections collected at different Z-axis depths. Deparaffination and rehydration with xylene and a graded series of ethyl alcohols (from 100% to 50%), antigen retrieval, primary antibody incubation, antigen visualization, and section mounting were performed as described above. Four images at 10× magnification were captured from each slice and examined for semi-quantitative analysis as disclosed above.

#### 2.3.7. Statistical Analysis

The data are expressed as the mean ± standard deviation. The experimental data for TGF-β1 expression in human bone ECM were analysed by GraphPad Prism 7 software (https://www.graphpad.com/scientific-software/prism/, accessed on 10 September 2018). The Mann–Whitney test was used to compare the groups.

## 3. Results and Discussion

### 3.1. TGF-β1 Immunolocalization and Quantification in Bone ECM

TGF-β1 is commonly stored in bone ECM as an inactive form, combined in a complex that enhances protein stability. During the ECM resorption phase, TGF-β1 is released and activated, becoming able to exert several functions on bone biology [4,8,9,10,11,12,13].

Results from immunohistochemical and enzyme-linked immunosorbent assay (ELISA) analyses on bone tissue samples were exploited as key information to define the subsequent encapsulation of TGF-β1 into PLGA NPs and their dispersion into a collagen-based formulation, to develop a 3D printed scaffold mimicking the GF bone content and distribution.

**Figure 3:**
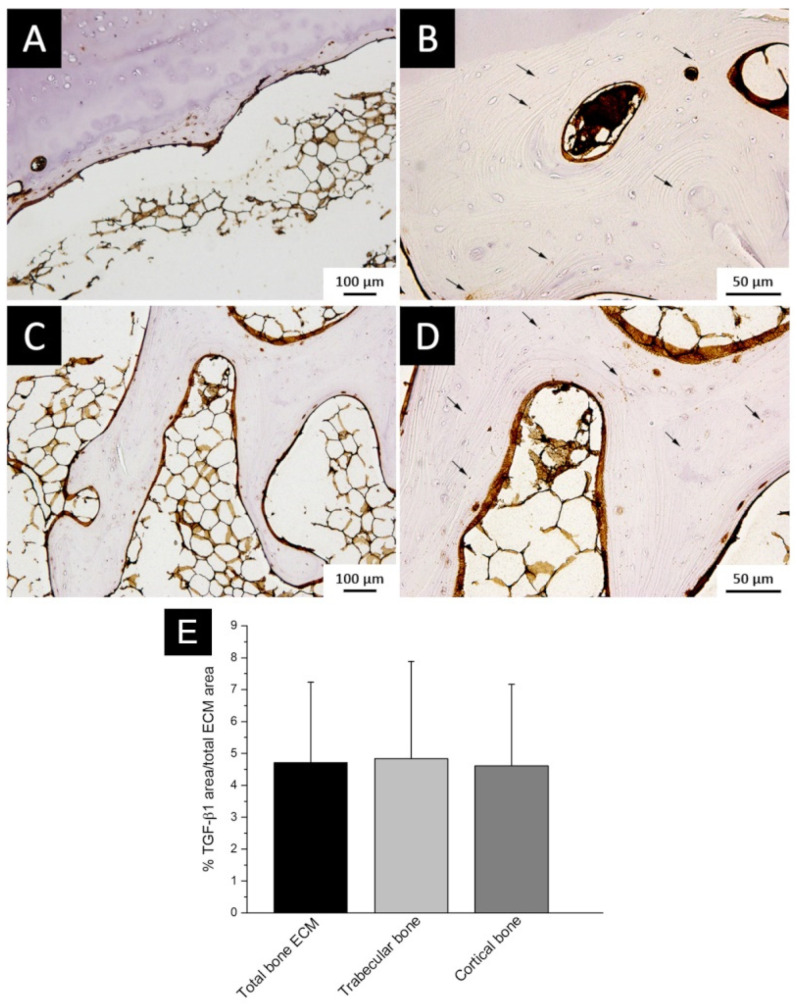
TGF-β1 staining in bone tissue and semi-quantitative analysis (**A**–**D**): TGF-β1 staining in cortical (**A**,**B**) and trabecular (**C**,**D**) bone tissue. ((**A**,**C**) 10× magnification; (**B**,**D**) 20× magnification); black arrows highlight the protein accumulation in bone ECM. (**E**) Histogram representing area percentage of TGF-β1 staining.

The localization of TGF-β1 stored in bone ECM, evaluated by immunohistochemistry, showed that the staining was mainly displayed as abundant spots of TGF-β1, corresponding to stored protein accumulations, homogenously distributed (Figure 3B,D). Furthermore, a weak TGF-β1 expression along lamellae and in osteocyte lacunae was also observed and as expected, a higher positivity was detected throughout the endosteum (Figure 3A–D).

Immunostaining quantification to calculate the percentage of area distribution showed that TGF-β1 covered the 4.47 ± 3.03% of the total bone ECM area, while no significant differences between trabecular and cortical bone (trabecular bone 4.83 ± 3.05% vs cortical bone 4.62 ± 2.55%, *p* = 0.93) were detected (Figure 3E). These findings suggested that most of the stored TGF-β1 is mainly distributed in numerous crowds, equally disseminated in ECM of both trabecular and cortical bone.

Furthermore, the TGF-β1 quantity in protein extract obtained from 1 g of decellularized bone tissue sample has been analysed to assess the effective protein amount present in the ECM. The ELISA assay detected 258.2 ± 30.2 pg of TGF-β1/g of bone.

TGF-β1 spots stain and quantification were taken into account for the subsequent design of a biomimetic 3D printed scaffold, able to mimic what was observed in human bone.

### 3.2. Characterisation of PLGA_TGF-β1 Nanoparticles (NPs)

PLGA NPs containing TGF-β1 (PLGA_TGF-β1) were prepared by a double emulsion solvent evaporation method exploiting poly(vinyl alcohol) (PVA) as a stabilizer in the aqueous phase. The resulting nanocarriers were characterized in terms of morphology, size distribution and surface charge.

**Figure 4:**
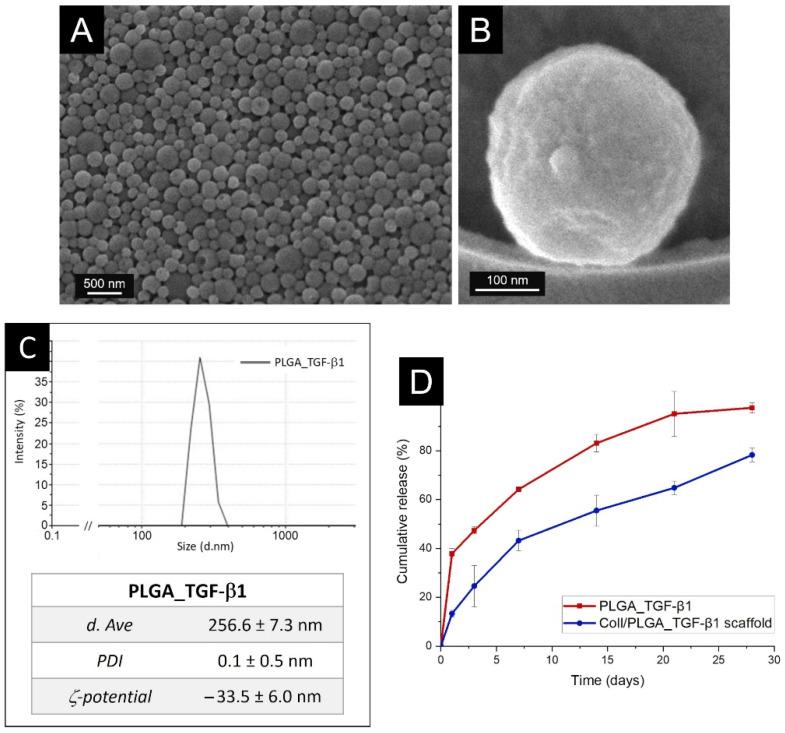
PLGA_TGF-β1 nanoparticles characterisation: FESEM images at different magnifications: 25,000× (**A**) and 200,000× (**B**) and DLS size distribution graph and numerical results (**C**) (abbreviations: d.Ave: average diameter, PDI: polydispersity index). (**D**) TGF-β1 release profile from PLGA nanoparticles (red line) and composite scaffold (blue line) under physiological conditions (PBS, 37 °C, pH 7.4).

Field-Emission Scanning Electron Microscopy (FESEM) observation showed that PLGA_TGF-β1 NPs were mostly homogeneously dispersed as individual particles, with a well-defined spherical structure and a smooth surface, presenting a diameter of about 250 nm (Figure 4A,B).

The particle size distribution was evaluated by Dynamic Light Scattering (DLS) and the obtained results demonstrated uniform-sized PLGA_TGF-β1 NPs (Figure 4C), with an average diameter of 256.6 ± 7.3 nm, confirming the morphological assessment by FESEM. Besides, the developed NPs exhibited a narrow and monodispersed size distribution and a polydispersity index (PDI) lower than 0.1 (i.e., 0.1 ± 0.5), in full accordance with FESEM observation. Moreover, the GFs-loaded polymeric nanocarriers showed a negative zeta potential value, lower than −30 mV (−33.5 ± 6.0 mV), proving good colloidal stability when suspended in bi-distilled water (ddH_2_O) [79]. The zeta potential of NPs, besides representing a key factor for their suspension stability, influences the adsorption phenomena onto the material surface, and consequently the biodistribution in vivo as well as the interaction with the cell membrane [80]. On the contrary, results showed that not-loaded PLGA nanoparticles (PLGA_Ø) possessed an almost neutral surface charge when suspended in ddH_2_O (−0.3 ± 0.1 mV). These results suggest that the negative potential of loaded PLGA NPs can be ascribed to the exposure also on the carrier surface of encapsulated TGF-β1, whose amino acids are characterised by a negative charge at physiological pH [81].

The encapsulation efficiency (EE%) of TGF-β1 into the nanoparticles was determined by ELISA assay after the dissolution of PLGA matrix by using 0.1 M NaOH containing 0.5% (*w*/*v*) Sodium Dodecyl Sulphate (SDS). The EE% was registered to be 63.93 ± 7.91%, resulting in an actual loading concentration of about 1.28 ng TGF-β1 per mg of nanoparticles. An alternative method has also been exploited to estimate indirectly the efficiency of encapsulation into the nanocarriers by measuring the amount of non-encapsulated biomolecules in the aqueous supernatants recovered after washing. The indirect encapsulation efficiency (iEE%), obtained by calculating the difference between the initial amount of GFs used for the incorporation and the amount of TGF-β1 detected in the washing medium [82], resulted to be 80.05 ± 5.62%. The discrepancy of the obtained results can be attributed to the lysis method used for the direct quantification of TGF-β1 that was entrapped into PLGA NPs, as most likely a fraction of GFs was lost or damaged during the dissolution step of the PLGA matrix. The employed procedure, necessary for the extraction of the encapsulated biomolecules, implied the dissolution of the polymeric network with a surfactant-added alkaline medium in which the biomolecules precipitated allowing the recovery. Indeed, the alkaline medium can affect the biomolecule structure and therefore be detrimental to the overall functionality [83,84]. Moreover, among the challenges associated with this method, the partial precipitation of the incorporated biomolecules is commonly reported and can be also invoked as one of the reasons for the different values of EE% and iEE% registered during the analysis [85].

### 3.3. In Vitro TGF-β1 Release Kinetics from PLGA Nanoparticles

A suitable release profile represents one of the most important characteristics of a DDS, allowing the adequate biological action of the encapsulated cargo at the appropriate time. The in vitro release study of PLGA_TGF-β1 was carried out at physiological conditions (Phosphate Buffered Saline (PBS), 37 °C, pH 7.4) up to 28 days and the resulting kinetics, result of the combination of polymer degradation and biomolecule diffusion throughout the polymer network [44], is reported in Figure 4D. In detail, an initial burst release of 37.83 ± 2.12% of the initial amount of incorporated TGF-β1 was observed in the first 24 h, which can be ascribed to the protein adsorbed on the surface of the nanocarriers. After 1 day of incubation, TGF-β1 was progressively released and the overall released proteins reached 97.57 ± 2.12% of the total incorporated amount after 28 days. The release profile of biomolecules from PLGA NPs is strongly influenced by the degradation mechanism of the co-polymeric material. As reported in different studies, the PLGA degradation mechanism is a combined process of surface and bulk diffusions, and surface and bulk erosions [43,86]. The diffusion of water inside the matrix induces the polymer hydrolysis into soluble oligomeric and monomeric products [86] and in turn creates paths for the protein diffusion and further polymer erosion until the complete nanoparticle dissolution: as the polymer degrades GFs are released into the surrounding environment. Biomolecules features also play an important role in attracting the aqueous phase into the matrix [87]. Particularly, the release kinetics is associated with the physico-chemical features and the solubility of the incorporated proteins, as well as polymer hydrophobicity [41,42].

### 3.4. Rheology Assessment of the Coll/PLGA_TGF-β1 System

**Figure 5:**
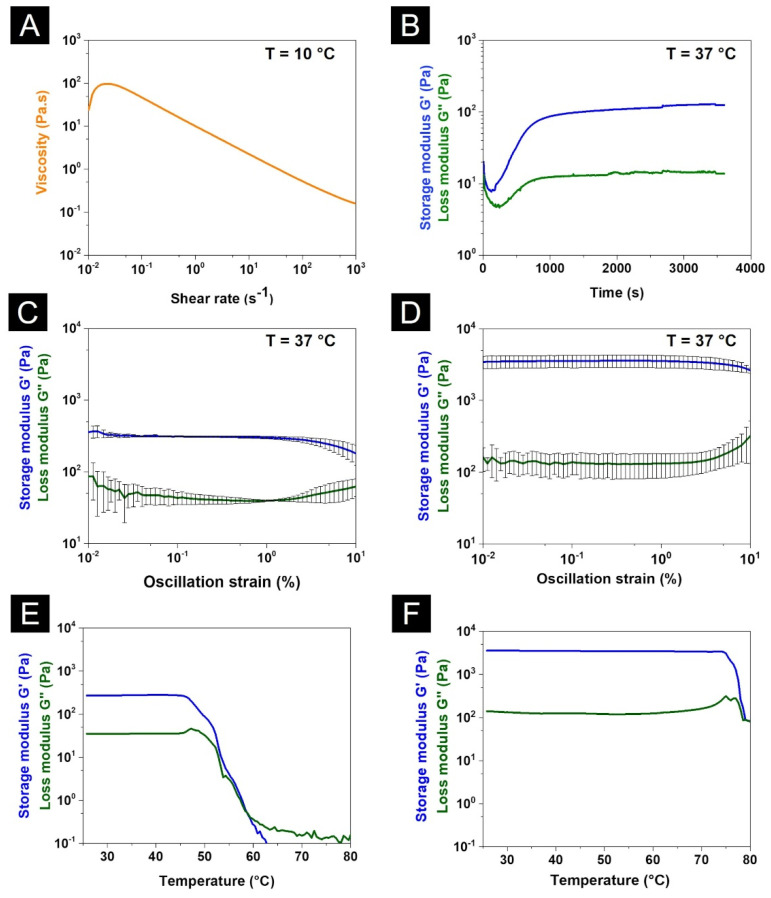
Rheological assessment of Coll/PLGA_TGF-β1 system: flow ramp at 10 °C (**A**) and sol–gel transition at 37 °C (**B**) of the Coll/PLGA_TGF-β1 system. Amplitude sweep tests at 37 °C (**C**,**D**) and temperature ramps (**E**,**F**) performed on Coll/PLGA_TGF-β1 before (**C**,**E**) and after (**D**,**F**) the chemical crosslinking with genipin.

The preliminary printability assessment of the developed hybrid formulation was obtained through rheological analyses investigating the variation of the material viscosity at increasing shear rates at 10 °C as well as its visco-elastic properties at 37 °C (Figure 5A,B). Moreover, the potential increase of the visco-elastic properties and denaturation temperature of the system after the chemical crosslinking with genipin was assessed by means of amplitude sweep tests and temperature ramps (Figure 5C–F). Figure 5A clearly showed the shear-thinning behaviour of the collagenous system, where an initial viscosity of about 86 Pa.s at lower shear rates rapidly decreases down to 0.14 Pa.s when a shear rate of 1000 s^−1^ is applied, proving the potential printability of the developed collagen-based material, according to different studies present in the literature [88].

The sol–gel transition of the system at 37 °C due to the collagen self-assembly was confirmed by the time sweep test reported in Figure 5B, where a stable gel was reconstituted after one hour with values of storage (G’) and loss (G’’) moduli of 124.8 Pa and 13.8 Pa, respectively.

These data showed that PLGA particles did not hinder the stability of the developed suspension at 10 °C as well as the natural reconstitution of the collagen matrix at 37 °C due to the assembly of collagen fibrils under simil-physiological conditions. However, even if the sol–gel transition of the system was induced after less than 200 s, a visible gap between G’ and G’’ and thus the reconstitution of a stable gel was observed after about 1000 s (Figure 5B). These results suggested the use of supporting material to avoid the collapse of the structure during the printing process [78,88] and hence a thermo-reversible gelatin support bath has been exploited during printing.

Considering the low mechanical properties of collagen-based materials, a chemical crosslinking method based on the use of genipin in ethanol solution was explored to improve the overall mechanical and thermal stability of the final composite system [17]. To this aim, gel samples of Coll/PLGA_TGF-β1 before and after reaction with genipin were studied exploiting amplitude sweep tests and temperature ramps, proving the effective chemical crosslinking of the material. The amplitude sweep tests were performed in a wide range of strains (0.01–10% strain) at 1 Hz and 37 °C, also identifying the visco-elastic region (LVR) of the material, while the temperature ramps investigated the variation of G’ and G’’ between 25 °C and 90 °C considering a ramp of 5 °C/min and a constant strain of 1% (inside the LVR previously identified by amplitude sweep test).

Figure 5C,D show the variation of G’ and G’’ in a wide range of strains applied to the material before and after the chemical crosslinking with genipin. In detail, the matrix obtained after the physical crosslinking of collagen (induced by physiological temperature and pH) led to G’ and G’’ values of about 296 Pa and 47 Pa respectively, indicating the successful reconstitution of a soft matrix. The linear visco-elastic region of the system was identified mainly in a range of strains between 0.1 and 1%. As expected, after genipin crosslinking, the value of G’ increased more than 10 times compared to the physically crosslinked system, measuring a mean value of about 3454 Pa. Furthermore, the increased strength and stability of the system were confirmed by the broadening of the LVR including almost the total range of strains between 0.01% and 10%.

Figure 5E,F show the temperature ramps performed to investigate the thermal stability of the system before and after the chemical crosslinking. The indicative denaturation temperature of the material was identified with the drop of both G’ and G’’ values. In detail, the denaturation temperature of Coll/PLGA_TGF-β1 increased from 45 °C up to 75 °C after the treatment with genipin. The significant increase in the material strength, as well as the denaturation temperature of the system, clearly suggested the successful chemical crosslinking and thus the formation of strong covalent bonds between collagen molecules. Based on the presented data, the chemical crosslinking with genipin was used after the printing process to stabilise and increase the mechanical properties of the scaffolds. The authors recently reported the high cytocompatibility of the genipin crosslinking method exploited to increase the mechanical properties and stability of the designed scaffolds, demonstrating the high viability and adhesion of MG-63 and Saos-2 cells onto genipin crosslinked collagen-based substrates [17].

### 3.5. Production of the 3D Printed Scaffolds

Based on the rheological properties of the developed formulations, the printing process was optimised to obtain mesh-like 3D structures. The scaffolds were obtained using an extrusion-based system and exploiting the FRESH (Freeform Reversible Embedding of Suspended Hydrogels) method reported by Hinton et al. [78] In detail, the use of a thermo-reversible gelatin support bath enables the deposition of 3D structures with improved printing fidelity when using biomaterial inks characterised by poor rheological properties. Once the structure is printed, the gelatin bath is melted by raising the temperature to 37 °C, thus enabling the post-processing of the printed constructs.

**Figure 6:**
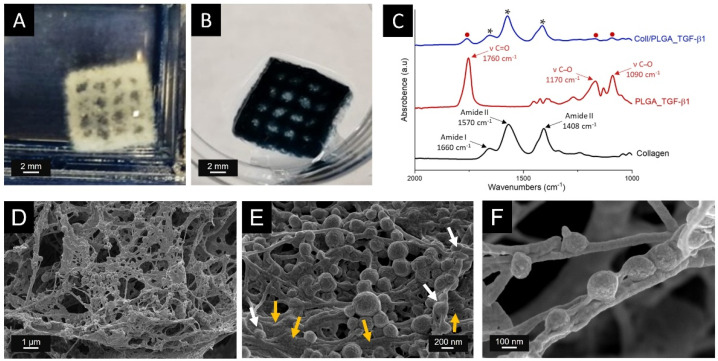
Coll/PLGA_TGF-β1 scaffolds characterisation: Optical images (**A**,**B**), ATR-FTIR spectra (**C**) and FESEM images (**C**–**F**) of 3D printed Coll/PLGA_TGF-β1 scaffolds. 3D mesh-like structure obtained by the optimised extrusion printing of Coll/PLGA TGF-β1 suspension before (**A**) and after (**B**) the genipin crosslinking treatment. (**C**) ATR-FTIR spectra of the different materials: collagen (black line), PLGA_ TGF-β1 nanoparticles (red line), Coll/PLGA_TGF-β1 (blue line, * collagen and • PLGA_ TGF-β1 peaks). FESEM images of Coll/PLGA_TGF-β1 scaffold at different magnification: 25,000× (**D**), 100,000× (**E**) and 250,000× (**F**). (**E**) Orange arrows indicate collagen fibrils properly reconstructed after incubation at 37 °C and white arrows highlight the successful embedding of PLGA_TGF-β1 nanoparticles into the collagen matrix.

According to the physico-chemical and rheological properties of the material ink, the best results in terms of printing fidelity were obtained setting pressure of 50 kPa and a printhead speed of 15 mm/s. 3D scaffolds having a surface of 10 mm × 10 mm and a thickness of about 1 mm were produced setting a layer thickness of 140 µm to promote the proper adhesion of subsequent layers. The 3D mesh-like scaffolds, shown in Figure 6A, were subsequently incubated at 37 °C for 3 h to induce the sol–gel transition of the system while removing the liquid gelatin bath.

To improve the strength and thermal stability of the scaffolds, the chemical crosslinking of collagen was induced by incubating the printed constructs in 0.5 wt% genipin in ethanol solution at 37 °C for 24 h. The binding of genipin to the tropocollagen molecule is the result of the nucleophilic substitution of the amino group of collagen on the olefinic carbon atom of genipin, followed by the opening of the dihydropyran ring to form the heterocyclic amine [89,90,91]. After crosslinking, the scaffolds were washed with distilled water to remove any residual of crosslinking agent and subsequently stored at 4 °C until testing.

### 3.6. Structural and Chemical Characterisation of Coll/PLGA_TGF-β1 Scaffolds

To explore the polymeric nanocarriers distribution into the collagenous matrix and the overall morphological features, the 3D printed mesh-like scaffolds were analyzed by means of FESEM. As shown in Figure 6D–F, cross-sectional FESEM images of the lyophilized composite scaffolds were acquired at various magnifications.

Images at lower magnifications (Figure 6D) displayed a uniform PLGA NPs distribution, without detecting significant particle agglomerates. The homogeneous dispersion of the GFs-nanocarriers can lead to a uniform TGF-β1 release from the scaffold when in contact with fluids while promoting the reinforcement of the collagen matrix [92]. In addition, the morphological assessment showed the fibrous structure of the collagenous matrix, proving the successful reconstruction of collagen fibrils after the incubation at 37 °C (Figure 6E). Higher magnification FESEM images also remarked the homogenous embedding of PLGA_TGF-β1 nanocarriers into the fibrillar nanostructure (Figure 6F).

Fourier transform infrared spectroscopy in attenuated total reflectance mode (ATR-FTIR) was performed to confirm the chemical features of the composite scaffold. The spectrum of Coll/PLGA_TGF-β1 reported in Figure 6C (blue line) exhibited the typical collagen peaks: the C=O stretching of amide I at 1660 cm^−1^, the N-H stretching of amide II at 1570 cm^−1^, and the C-N bending of amide III at 1408 cm^−1^ [93]. The characteristic absorption bands of PLGA were observed after the introduction of the nanocarriers into the collagenic matrix (Figure 6C, red spot). The absorption band at 1760 cm^−1^ was assigned to the C=O stretch vibration of copolymer ester bond while the weak band centred at 1170–1090 cm^−1^ was attributed to C-O stretch [94]. These data, combined with the morphological assessment by FESEM, confirmed the successful incorporation of PLGA NPs into the collagenous matrix. As expected, due to the limited amount of GFs compared to the amount of polymer, the PLGA_TGF-β1 spectrum is dominated by the vibrational bands ascribed to PLGA superimposed to the spectroscopic features of TGF-β1.

### 3.7. TGF-β1 Release Kinetics from the Composite Mesh-like Scaffold

The detection of the released amount of TGF-β1 from the composite scaffolds at different time points aimed at simulating the bone ECM environment in which the GFs are sustainably released during bone remodelling due to their homogenous distribution along and in the tissue layers. Accordingly, the release profile of TGF-β1 loaded NPs from the composite scaffold was monitored in PBS at 37 °C up to 28 days, reporting the cumulative percentage of released biomolecule as a function of time (Figure 4D). The initial burst release previously registered for the nanocarriers alone was significantly reduced when PLGA nanoparticles were incorporated into the collagenous matrix: 37.83 ± 2.12% and 13.29 ± 1.41% of TGF-β1 was released after 24 h of incubation from the PLGA NPs alone and the composite scaffold, respectively. The developed scaffold presented more sustained release kinetics, reaching 78.28 ± 2.83% after 28 days of incubation. These results indicated that the collagen matrix was able to modulate GFs release, confirming the proper embedding of PLGA NPs within the collagenous fibrous matrix. Interestingly, recent studies reported that a moderate initial burst followed by a sustained release of a biomolecule significantly improves the in vivo bone regeneration mechanisms [95,96]. A more gradual release of the cargo, favoured by the incorporation of loaded particles into different 3D polymeric matrices, was also observed in previous studies reported in the literature and by the authors [52,53,56,67]. Moreover, the biocompatibility of PLGA used as delivery systems of biomolecules, as well as bovine type I collagen, largely exploited as 3D support for cells, was widely demonstrated both in in vitro and in vivo studies reported in the literature [17,97,98].

### 3.8. Histochemical Assessment of PLGA_TGF-β1 Nanoparticle Distribution into the Collagen-Based Scaffold

**Figure 7:**
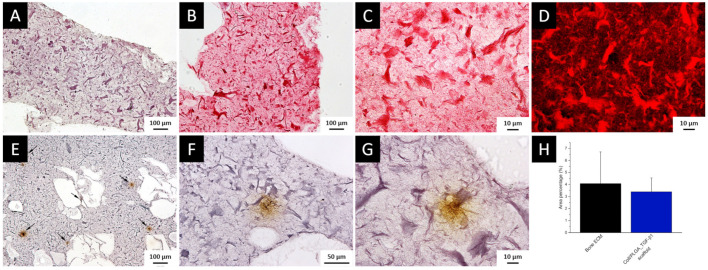
Histological staining in 3D-printed scaffolds (**A**–**D**): (**A**) Haematoxylin and Eosin staining; (**B**–**D**) Sirius Red staining underlining collagen fibres observed by brightfield microscope (**B**,**C**) and fluorescence microscope (**D**) ((**A**,**B**) 10× magnification; (**C**,**D**) 40× magnification). TGF-β1 staining in 3D-printed scaffold and semi-quantitative analysis (**E**–**H**): TGF-β1 staining representing PLGA_ TGF-β1 NPs inclusion in the scaffold structure at different magnification: 10× (**E**); 20× (**F**); 40× (**G**); (**E**) black arrows highlight the protein accumulation inside PLGA_TGF-β1 NPs throughout the collagen-based scaffold. (**H**) Histogram representing area percentage of TGF-β1 staining in 3D-printed scaffold measured by semi-quantitative analysis.

To investigate the distribution of collagen fibres in the 3D printed scaffolds, two different histological stains were performed, i.e., the Hematoxylin and Eosin staining combined with the Sirius Red staining, which binds specifically collagen molecules(Figure 7A–D, respectively). Histological analyses showed collagen fibres well-distributed all along the 3D printed meshes, and Sirius Red staining highlighted the presence of uniformly disseminated collagen aggregates (Figure 7C,D).

To compare the distribution of PLGA_TGF-β1 NPs throughout the scaffolds with the protein staining observed in human bone tissue samples, immunohistochemistry analysis was conducted on the 3D printed scaffolds. Results showed that PLGA_TGF-β1 NPs were homogeneously dispersed within the 3D printed constructs (Figure 7E–G, brown stain). Differently from bone ECM, where TGF-β1 was distributed as numerous small spots (Figure 3D), in the 3D printed scaffold the protein appeared as large aggregates homogeneously dispersed along the structure. This discrepancy can be ascribed to the concentration of TGF-β1 inside nanometric PLGA carriers since the GF was not directly dispersed into collagenous suspension to avoid its rapid release. Although a uniform protein distribution throughout the structure has been achieved thanks to the printing of the developed collagen-based hybrid formulation with a single printing head, as a future perspective to provide a more biomimetic localization of the GF into the collagenous construct, two separate printing heads can be exploited to deliver the polymeric ink and the PLGA_TGF-β1 NPs suspension.

Although the size of TGF-β1 aggregates appeared different in the two investigated systems (bone ECM and 3D printed structure), immunostaining quantitation revealed that TGF-β1 covered 3.39 ± 1.15% of scaffold area, showing a comparable percentage of area distribution with that detected in bone ECM (*p* = 0.99) (Figure 7H). The overall results proved that the collagenous system incorporating PLGA_TGF-β1 nanocarriers successfully mimicked the TGF-β1 content present in human bone tissue, also achieving a homogenous distribution of the GF in the scaffold.

## 4. Conclusions

Common limitations of the use of growth factors (GFs) in regenerative medicine approaches are linked to their short period of biological activity as well as the appropriate local effective concentrations, which are essential for tissue regeneration. The use of GFs for clinical application could therefore take advantage of the design of smart delivery platforms.

In this context, the present study demonstrated the successful development of polymeric PLGA nanocarriers able to encapsulate and gradually release TGF-β1 (PLGA_TGF-β1) as promising tools for the design of devices for bone regeneration. A further goal of the study was the design of a 3D composite scaffold based on type I collagen and PLGA nanoparticles (Coll/PLGA_TGF-β1) able to mimic the bone ECM in terms of TGF-β1 distribution. To these aims, immunohistochemical and ELISA analyses were performed to localize and quantify the amount of TGF-β1 present in human bone ECM samples. The resulting scaffolds showed the capability to properly incorporate GFs loaded-PLGA nanocarriers providing a more sustained release of TGF-β1 compared to that detected from PLGA nanocarriers alone.

To date, no studies have reported the design of biomimetic collagen-based constructs embedding PLGA nanoparticles for the release of TGF-β1 with a GFs amount and distribution comparable to those observed in natural human bone ECM. The use of scaffolds containing nanocarriers able to release therapeutic biomolecules can represent a great advantage in the field of drug delivery systems (DDS), thanks to the opportunity not only to obtain local delivery but also a long-term therapeutic activity. In particular, the developed composite scaffolds can improve the in vivo stability of the delivered biomolecules, decrease their potential toxicity, prolong their residence time in situ and allow the combination with other active agents or supporting excipients. Future cellular in vitro studies will aim at investigating the activity preservation of TGF-β1 after release and evaluating the influence of the available GF on human osteoblasts and osteoclasts crosstalk.

## Data Availability

The data is accessible upon request from the corresponding author.
